# The impact of antibiotic induction on virulence and antibiotic resistance in *Klebsiella pneumoniae*: a comparative study of CSKP and CRKP strains

**DOI:** 10.3389/fmicb.2024.1498779

**Published:** 2024-10-21

**Authors:** Ke-Da Chen, Wei Chen, Qian Zhang, Qingcao Li

**Affiliations:** ^1^Department of Blood Transfusion, The Affiliated LiHuiLi Hospital of Ningbo University, Ningbo, China; ^2^Department of Clinical Laboratory, The Affiliated LiHuiLi Hospital of Ningbo University, Ningbo, China

**Keywords:** *Klebsiella pneumoniae*, carbapenem-sensitive, carbapenem-resistant, virulence gene, resistance gene

## Abstract

**Background:**

*Klebsiella pneumoniae* is an opportunistic pathogen causing nosocomial infections, classified into carbapenem-sensitive and carbapenem-resistant strains. Understanding the virulence factors and antibiotic resistance of these strains is essential for effective clinical management.

**Objective:**

This study compared the virulence genes and antibiotic resistance profiles of 50 CSKP and 50 CRKP strains, examining their expression under antibiotic pressure and the mechanisms contributing to their pathogenicity.

**Methods:**

Virulence genes (*rmpA*, *rmpA2*, *iucA*, *iutA*, *Peg-344*, *ybts*, *iroB*) were detected in both strains using polymerase chain reaction (PCR). Antibiotic susceptibility testing established minimum inhibitory concentrations (MICs) for key antibiotics. Gene expression analysis was performed with quantitative reverse transcription PCR (qRT-PCR) after 10 days of antibiotic exposure.

**Results:**

CSKP strains exhibited significantly higher positivity rates for virulence genes compared to CRKP strains. CRKP strains predominantly expressed resistance genes *KPC*, *SHV*, and *CTX-M3*, whereas no resistance genes were found in CSKP. Antibiotic susceptibility tests showed increased MICs, particularly for ciprofloxacin and imipenem, following antibiotic induction. CSKP demonstrated elevated expression of *rmpA* and *rmpA2*, while CRKP showed increased expression of *SHV*, and *KPC* after antibiotic exposure.

**Conclusion:**

This study highlights the intricate relationship between virulence and resistance in *Klebsiella pneumoniae*. CSKP strains show strong virulence factor expression, while CRKP strains adapt to antibiotic pressure through altered gene expression patterns. These findings underscore the urgent need for continuous surveillance and innovative therapeutic strategies to combat multidrug-resistant *Klebsiella pneumoniae* infections.

## 1 Introduction

*Klebsiella pneumoniae* is a Gram-negative bacterium belonging to the Enterobacteriaceae family, recognized as a significant opportunistic pathogen responsible for a range of infections, including pneumonia, septicemia, and urinary tract infections (Choby et al., 2020). This organism primarily resides in the human gastrointestinal tract but poses a serious risk, particularly to immunocompromised individuals (Kar et al., 2023; Kuo et al., 2022). Its notorious ability to rapidly acquire antibiotic resistance has made it a leading cause of nosocomial infections globally (Abbas et al., 2024; Uddin et al., 2021).

The emergence of multidrug-resistant *Klebsiella pneumoniae* strains presents an escalating public health challenge, contributing to extended hospital stays and heightened mortality rates. A key factor influencing the pathogenicity of this bacterium is its array of virulence factors alongside its antibiotic resistance genes (Assefa, 2022). Notable virulence determinants include the production of polysaccharide capsules, adhesins, and various tissue-damaging enzymes. The capsule plays a crucial role in defending against phagocytosis by the immune system (Riwu et al., 2022; Gao et al., 2024). Additionally, siderophores are vital for iron acquisition, which is critical for bacterial survival during infection (Khasheii et al., 2021). Compounding the challenges in treating *Klebsiella pneumoniae* infections is the presence of antibiotic resistance genes, such as bla*KPC*, which confers resistance to carbapenems, and those associated with extended-spectrum beta-lactamases (ESBLs), complicating treatment efforts (Aminov, 2009; Denk-Lobnig and Wood, 2023).

A relatively recent area of research is the effect of antibiotic exposure on the expression of both virulence and resistance genes in *Klebsiella pneumoniae*. As antibiotics impose selective pressure, understanding how sub-lethal antibiotic concentrations may induce gene expression changes that enhance virulence or promote resistance acquisition has become increasingly important (Andersson and Hughes, 2014; Sanchez-Cid et al., 2023; Li et al., 2019).

As our knowledge of *Klebsiella pneumoniae* evolves, studying the consequences of antibiotic-induced changes in gene expression is essential for developing effective therapeutic strategies. It is crucial to explore how integrating antibiotics into treatment regimens may inadvertently enhance the pathogenicity of these organisms.

In this study, we examined the alterations in the expression of virulence and antibiotic resistance genes in *Klebsiella pneumoniae* following exposure to various classes of antibiotics. Using quantitative real-time PCR, we assesed the expression levels of key virulence factors and resistance genes under different antibiotic conditions. Our findings aim to deepen the understanding of how *Klebsiella pneumoniae* adapts to antibiotic pressure and to inform clinical practices regarding antibiotic use, especially in environments where this pathogen poses a significant threat to patient safety. By exploring the dynamics of virulence and resistance under antibiotic pressure, we hope to contribute valuable insights for prevention and treatment strategies against infections caused by this formidable pathogen.

## 2 Materials and methods

### 2.1 Bacterial strains

This study involved the selection of 100 *Klebsiella pneumoniae* strains, comprising fifty carbapenem-sensitive *Klebsiella pneumoniae* (CSKP) strains and fifty carbapenem-resistant *Klebsiella pneumoniae* (CRKP) strains. These strains were retrieved from a preserved culture collection at the microbiology laboratory of Ningbo Medical Center LiHuiLi Hospital.

The identification of the bacterial strains and their antimicrobial susceptibility assessment were conducted using the VITEK 2 Compact automated system (bioMérieux, France). For detailed information on the susceptibility profiles of these strains, please refer to Supplementary Table 1.

To validate the antibiotic susceptibility testing procedures, control strains of *Escherichia coli* ATCC 25922 and *Pseudomonas aeruginosa* ATCC 27853 were included as benchmarks in the experiments. These control strains were sourced from the National Center for Clinical Laboratories, Ministry of Health. The research protocol was approved by the Ethics Committee of Ningbo Medical Center LiHuiLi Hospital, under the approval number (KY2024SL286-01), ensuring adherence to ethical guidelines in medical research.

### 2.2 Selection and identification of bacteria

We performed conventional PCR to detect virulence and antimicrobial resistance genes in bacterial strains. DNA was extracted by lysing cells in a buffered solution and heating at 100°C for 10 min. After centrifugation at 12,000 rpm for 5 min, the supernatant containing approximately 100 ng/μL of DNA was collected.

The PCR reaction consisted of 2.0 μL of extracted DNA, 1.0 μL each of forward and reverse primers, 12.5 μL of 2X Taq MasterMix (Thermo Fisher Scientific, USA), and 8.5 μL of double-distilled water, totalling 25 μL. Thermal cycling included an initial denaturation at 94°C for 3 min, followed by 30 cycles of denaturation at 94°C for 30 s, annealing at 55°C for 30 s, and extension at 72°C for 1 min, concluding with a final extension at 72°C for 10 min. Amplified products were analyzed using agarose gel electrophoresis, selecting eight carbapenem-sensitive and eight carbapenem-resistant strains for further analysis.

### 2.3 Antibiotic induction experiment

Following the PCR results, eight strains of CRKP, which contained both resistance genes and virulence genes, were selected for the antibiotic induction experiment. Additionally, eight strains of CSKP which possessed the same virulence genes, were also identified for further study.

To culture the selected bacterial strains, they inoculated into LB broth and incubated at 37°C until they reached the logarithmic growth phase. The experimental protocol induced by antibiotics has been slightly adjusted (Wistrand-Yuen et al., 2018). The induction schedule was established as follows: the strains were exposed to an initial concentration of 1/2 the minimum inhibitory concentration (MIC) of each antibiotic on Day 1. The concentration was increased to 1 MIC on Day 2, followed by an increase to 2 MIC on Day 3. This gradual increase in antibiotic exposure continued for a total of 10 days, ultimately reaching 512 MIC, or the fixed concentration of 64 μg/mL if the initial MIC of the strain was greater than 64 μg/mL. During this entire period, bacteria were treated daily with the designated antibiotic concentrations, enabling the assessment of changes in antibiotic susceptibility and resistance patterns over time.

### 2.4 Real-time quantitative PCR (qPCR) analysis

After completing the antibiotic induction phase, three specific time points were selected for a detailed analysis: baseline strains prior to any exposure, as well as the strains induced on Day 4, Day 7, and Day 10 (Gaudin et al., 2013; Viers et al., 2014). To isolate total RNA from these cultures, the Column Bacterial Total RNA Extraction Purification Kit (Bioteke, Shanghai) was utilized according to the manufacturer’s instructions.

Following RNA extraction, reverse transcription was performed to generate complementary DNA (cDNA) from the isolated RNA samples. Subsequently, quantitative PCR (qPCR) was conducted using SYBR Green PCR Master Mix in conjunction with gene-specific primers, allowing for the measurement of expression levels of virulence and resistance genes both before and after antibiotic exposure. The qPCR protocol involved an initial denaturation at 95°C for 3 min, followed by 40 cycles of denaturation at 95°C for 3 s and annealing at 60°C for 30 s.

The specific primer sequences for each gene were listed in Supplementary Table 2. Additionally, each gene was analyzed in triplicate to ensure the reliability and accuracy of the results. For the purpose of quantitative analysis, the 16S rRNA gene was employed as an internal control, and the data were quantified using the 2^−ΔΔCT^ method for expression analysis.

### 2.5 Statistical analysis

Statistical analyses were performed using SPSS version 26.0. To assess the differences in gene expression between the original strains and the induced strains at Day 4, Day 7, and Day 10, one-way ANOVA was utilized, complemented by Dunnett’s test for multiple comparisons. Results are expressed as mean values ± standard deviations (SD). A significance threshold of *p* < 0.05 was established to identify statistically significant differences.

Graphical representation of the data was performed using GraphPad Prism version 10.1.2. The software was utilized to create bar graphs illustrating the mean expression levels of virulence and resistance genes at each time point. The error bars represented standard deviations. Statistical significance was marked with asterisks based on the results of the Dunnett’s test. The combination of SPSS for statistical analysis and GraphPad Prism for visualization provided a comprehensive overview of the impacts of antibiotic exposure on gene expression in *Klebsiella pneumoniae*.

## 3 Results analysis

### 3.1 Detection of virulence and resistance genes

In this study, the antimicrobial susceptibility testing conducted on 100 isolates indicated that CRKP demonstrates a high resistance rate to most cephalosporins, with a resistance rate reaching 46% for both amikacin and cotrimoxazole. In contrast, CSKP showed a resistance rate to cephalosporins ranging from 10 to 30%, and was highly susceptible to both imipenem and ertapenem. We analyzed these strains for the presence of virulence genes, with an emphasis on examining 50 CRKP strains for resistance genes. Among the 50 CSKP strains, we determined the positivity rates for seven virulence genes: *rmpA*, *rmpA2*, *iucA*, *iutA*, *Peg-344*, *ybts*, and *iroB*. The findings indicated that the positive rates were as follows: *rmpA*: 50.0%, *rmpA2*: 46.0%, *iucA*: 52.0%, *iutA*: 42.0%, *Peg-344*: 54.0%, *ybts*: 54.0%, and *iroB*: 50.0%. In comparison, the 50 CRKP strains exhibited the following positivity rates for the same virulence genes: *rmpA*: 12.0%, *rmpA2*: 46.0%, *iucA*: 48.0%, *iutA*: 50.0%, *Peg-344*: 2.0%, *ybts*: 20.0%, and *iroB*: 82.0%. Notably, the positive detection rates for *rmpA*, *iroB*, *Peg-344*, and *ybts* in CSKP strains were significantly higher than those observed in CRKP strains (*P* < 0.05). However, no statistically significant differences were noted for the other virulence genes (*P* > 0.05).

Regarding the resistance genes in the CRKP strains, the detection rates were as follows: *KPC*: 86.0%, *SHV*: 94.0%, and *CTX-M3*: 38.0%. Remarkably, no resistance genes were identified in any of the CSKP strains (see Table 1 for detailed results).

**TABLE 1:** Detection rate of virulence and resistance genes.

Gene type	Gene name	CRKP [*n* (%)]	CSKP [*n* (%)]	*P*
Virulence genes	*rmpA*	6 (12.0)	25 (50.0)	<0.001
	*rmpA2*	23 (46.0)	23 (46.0)	1.000
	*iucA*	24 (48.0)	26 (52.0)	0.689
	*iutA*	25 (50.0)	21 (42.0)	0.422
	*iroB*	1 (2.0)	27 (54.0)	<0.001
	*Peg-344*	10 (20)	27 (54.0)	<0.001
	*ybts*	41 (82.0)	25 (50.0)	<0.001
Resistance genes	*KPC*	43 (86.0)	/	/
	*Ndm*	7 (14.0)	/	/
	*CTX-M-1*	0 (0)	/	/
	*CTX-M3*	19 (38.0)	/	/
	*SHV*	47 (94.0)	/	/
	*DHA*	2 (4.0)	/	/

### 3.2 Antibiotic induction outcomes

From the initial 50 strains of CSKP and 50 strains of CRKP, we selected eight representative strains from each group for further analysis. This selection was based on a criterion that aimed to encompass a diverse range of virulence and resistance gene profiles, ensuring that the selected strains collectively reflected the genetic variability present in the broader sample. The specific virulence and resistance gene distribution among these strains can be found in Supplementary Table 3. In this experiment, we evaluated the Minimum Inhibitory Concentrations (MICs) of *Klebsiella pneumoniae* against various antibiotics, both before and after a 10-day antibiotic induction period. The initial MIC values are documented in Supplementary Table 4.

**FIGURE 1:**
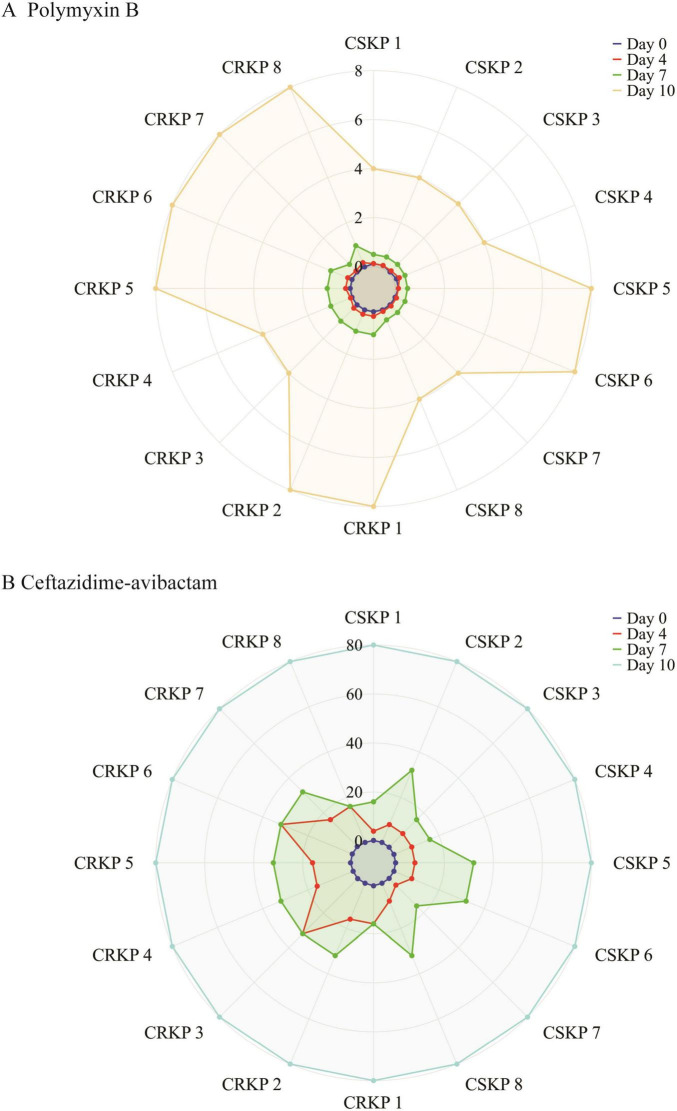
**(A)** Radar chart of *Klebsiella pneumoniae* induced by polymyxin B. **(B)** A radar chart of *Klebsiella pneumoniae* induced byceftazidime-avibactam. The areas enclosed at each time point represent the antibiotic resistance status of the bacteria at that time.

By Day 4, we observed that the MICs for ciprofloxacin and imipenem escalated rapidly, exceeding 64 μg/mL. In contrast, the MIC for ceftazidime-avibactam ranged between 4 and 32 μg/mL, while polymyxin B exhibited an MIC between 0.125 and 0.5 μg/mL. By Day 7, the MIC for ceftazidime-avibactam increased to between 16 and 32 μg/mL, and the MIC for polymyxin B rose to between 0.5 and 1 μg/mL. On Day 10, the MIC for ceftazidime-avibactam surpassed 16 μg/mL, while the MIC for polymyxin B ranged from 4 to 8 μg/mL. The detailed induction results of polymyxin B and ceftazidime-avibactam are illustrated in Figure 1.

### 3.3 Gene expression analysis via qPCR

The results from conventional PCR and the antibiotic induction experiments highlighted the need to quantitatively assess the expression levels of virulence and antimicrobial resistance genes. Therefore, we employed real-time quantitative PCR (qPCR) for a more precise measurement of gene expression. Within the CSKP and CRKP groups, we selected the virulence genes *rmpA*, *rmpA2*, *iucA*, and *iutA*, which exhibited high positive rates. Additionally, for the CRKP strains, we included the resistance genes *SHV* and *KPC*, which also demonstrated significant positive rates, for further analysis. The qPCR results indicated that there were significant differences in the expression levels of virulence and resistance genes before and after antibiotic induction.

**FIGURE 2:**
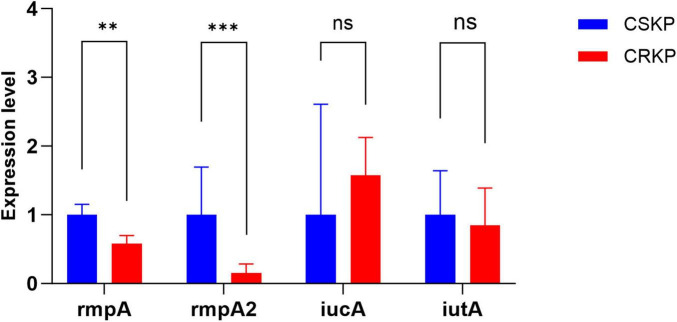
Differential gene expression level of rmpA, rmpA2, iucA and iutA in CSKP and CRKP. Using CSKP as a control, the differences in gene expression level of CRKP in various genes are compared. ***P < 0.001, **P < 0.01, and “ns” represents no statistically significant difference between the two groups.

Initially, the expression levels of the virulence genes *rmpA* and *rmpA2* in the original carbapenem-sensitive strain (CSKP) were found to be significantly higher compared to those in CRKP (*P* < 0.05). However, the expression levels of *iucA* and *iutA* showed no significant differences between the two groups (Figure 2).

**FIGURE 3:**
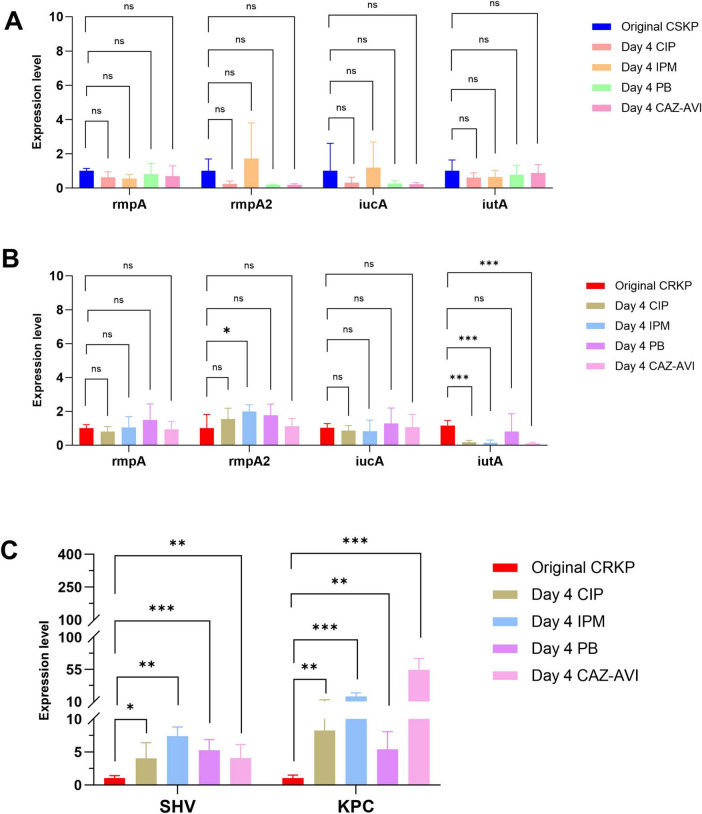
Differential gene expression level in CSKP and CRKP on Day 4. Using CSKP in panel **(A)** as a control, the differences in gene expression level of rmpA, rmpA2, iucA and iutA in CSKP are compared. Using CRKP in panels **(B,C)** as a control, the differences in gene expression level of rmpA, rmpA2, iucA, iutA, SHV, KPC in CRKP are compared. ***P < 0.001, **P < 0.01,*P < 0.05, and “ns” represents no statistically significant difference between the two groups.

After 4 days of antibiotic induction, the virulence gene expression in CSKP remained relatively stable. Conversely, in CRKP, the expression of the *iutA* gene significantly decreased when compared to the original CRKP strain (*P* < 0.05). Notably, the expression levels of the resistance genes *SHV* and *KPC* in CRKP showed a significant increase (Figure 3).

**FIGURE 4:**
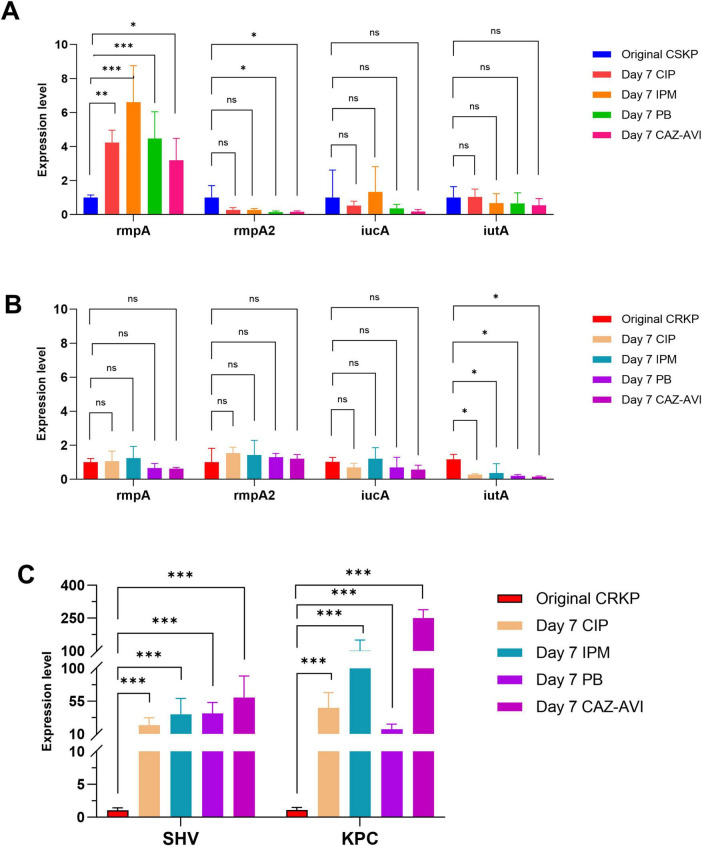
Differential gene expression level in CSKP and CRKP on Day 7. Using CSKP in panel **(A)** as a control, the differences in gene expression level of rmpA, rmpA2, iucA and iutA in CSKP are compared. Using CRKP in panels **(B,C)** as a control, the differences in gene expression level of rmpA, rmpA2, iucA, iutA, SHV, KPC in CRKP are compared.***P < 0.001, **P < 0.01, *P < 0.05, and “ns” represents no statistically significant difference between the two groups.

Following 7 days of antibiotic induction, the expression of the *rmpA* gene in CSKP significantly increased, whereas the expression levels of the other three genes demonstrated minimal changes. In CRKP, the expression of *iutA* still decreased (*p* < 0.05) alongside a notable increase in the expression levels of resistance genes (*P* < 0.001) (Figure 4).

**FIGURE 5:**
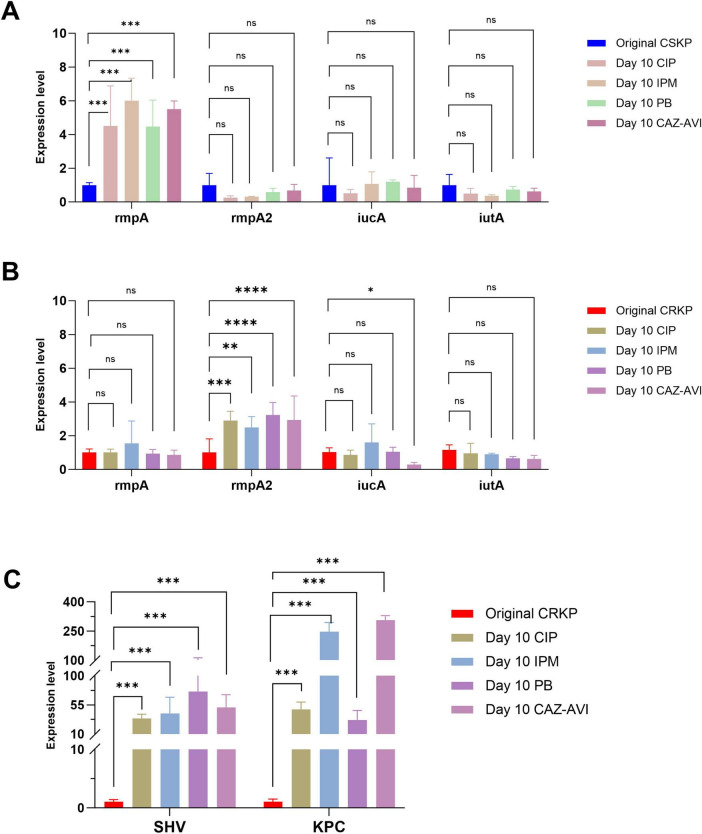
Differential gene expression level in CSKP and CRKP on Day 10. Using CSKP in panel **(A)** as a control, the differences in gene expression level of rmpA, rmpA2, iucA and iutA in CSKP are compared. Using CRKP in panels **(B,C)** as a control, the differences in gene expression level of rmpA, rmpA2, iucA, iutA, SHV, KPC in CRKP are compared. ****P < 0.0001, ***P < 0.001, **P < 0.01, *P < 0.05, and “ns” represents no statistically significant difference between the two groups.

Finally, after 10 days of antibiotic induction, the expression of the *rmpA* gene in CSKP was higher in comparison to the original strain but showed minimal change relative to the strain induced for 7 days. In contrast, the expression of *iutA* in CRKP shifted from being statistically significant compared to the original CRKP strain to no longer demonstrating significant differences. Nonetheless, the expression of *rmpA2* exhibited a marked increase compared to the original CRKP strain, yielding a statistically significant difference, while the levels of resistance gene expression continued to rise significantly (Figure 5).

## 4 Discussion

*Klebsiella pneumoniae* is a significant pathogen responsible for a wide range of infections, particularly in immunocompromised patients. In recent years, there has been increasing concern regarding its rising antibiotic resistance. In the current susceptibility testing, CRKP was found to be resistant to most cephalosporin antibiotics, while CSKP exhibited resistance rates between 10 and 30%. Additionally, resistance to other antibiotics has also shown varying increases. Consequently, the presence of *Klebsiella pneumoniae* poses a significant challenge to our healthcare environment (Kot et al., 2023; Wysocka et al., 2021).

To conduct further analytical research, the detection of virulence genes provided additional insights into the pathogenic potential of CSKP and CRKP. In our study, we assessed a total of 100 strains of *Klebsiella pneumoniae* for seven virulence genes: *rmpA*, *rmpA2*, *iucA*, *iutA*, *Peg - 344*, *ybts*, and *iroB*. The virulence genes *rmpA* and *rmpA2* enhanced capsular polysaccharide production, helping *Klebsiella pneumoniae* evade the immune system (Cheng et al., 2010). The *iucA* and *iutA* genes facilitated iron acquisition through the synthesis of aerobactin and its receptor, promoting bacterial growth in iron - limited conditions (Burbank et al., 2015). Additionally, *Peg - 344* and *ybts* were involved in enhancing virulence and biofilm formation, while *iroB* was part of the siderophore gene cluster that aided in iron uptake (Hwang et al., 2024; Hogle et al., 2022). Together, these genes significantly contributed to the pathogenicity of *Klebsiella pneumoniae*. Our study showed that the positive detection rates among CSKP were evidently high, particularly for *rmpA* and *rmpA2* at 50.0% and 46.0% respectively. In contrast, CRKP exhibited significantly lower rates, with *rmpA* at only 12.0%, although a remarkably higher positivity rate of *iroB* at 82.0% was observed. This pattern correlated with findings by Kochan et al. (2023), who emphasized the importance of virulence factors in determining pathogenicity within hypervirulent strains (Debroas and Siguret, 2019; Schroeder et al., 2017).

In terms of antibiotic resistance, the vast presence of resistance genes such as *KPC*, *SHV*, and *CTX-M3* in CRKP, with detection rates of 86.0%, 94.0%, and 38.0%, respectively, highlights an alarming trend of resistance among these strains. This aligns with observations by Pulingam et al. (2022) who correlated the increasing prevalence of resistance genes with the overuse of antibiotics in healthcare environments. *KPC* was particularly alarming as it conferred high - level resistance to carbapenems, often regarded as the last - line antibiotics for treating severe infections. The widespread detection of *SHV* and *CTX - M3* reinforced the challenge presented by extended - spectrum beta - lactamases (ESBLs), which could compromise the effectiveness of penicillins and cephalosporins (Yu et al., 2024).

The previous assessments of virulence and resistance genes aimed to provide a comprehensive evaluation of the pathogenicity and resistance profiles of the bacteria, thereby effectively guiding clinical treatment, monitoring disease trends, and controlling the spread of infections. Conversely, the drug induction experiments sought to explore the effects of antibiotics on bacteria, including the mechanisms of induced resistance, the identification of effective antimicrobial agents, and the adaptive changes of bacteria under antibiotic pressure. In this drug induction experiment, we selected eight representative strains from each of 50 CSKP and 50 CRKP isolates to ensure coverage of most virulence and resistance genes that required further investigation.

By using the agar dilution method to determine the minimum inhibitory concentration (MIC) of antimicrobial agents, we found that half of the original CRKP strains were resistant to ciprofloxacin and imipenem, while the other half exhibited sensitivity to these drugs, similar to CSKP strains. After 4 days of drug induction, all strains demonstrated resistance to ciprofloxacin and imipenem, with MIC values exceeding 64 μg/mL. As shown in Figure 1, after 4, 7, and 10 days of induction with ceftazidime-avibactam, the MIC values for both CSKP and CRKP increased from <0.5 to 32 μg, eventually surpassing 64 μg/mL. This indicates that prolonged exposure to antibiotics can lead to the development of multidrug resistance among these pathogens (Ching and Zaman, 2020; Stanton et al., 2020). However, it is noteworthy that after 10 days of induction with polymyxin B, the MIC changes for both CSKP and CRKP were more modest, shifting from <0.5 to around 8 μg. This minimal change may be attributed to the structural characteristics of polymyxin B itself. Because it exerted its lethal effect by interacting with the lipopolysaccharides of the bacterial outer membrane, disrupting its integrity. For bacteria to develop resistance, multiple genetic mutations would have needed to occur simultaneously to alter the outer membrane structure, which was a low-probability event (Yang et al., 2023).

To further compare the virulence and resistance of CSKP and CRKP after antibiotic induction, we conducted RT-qPCR to analyze the original strains as well as their antibiotic-induced counterparts. The analysis revealed that the expression levels of *rmpA* and *rmpA2* in CSKP strains were significantly higher than those in CRKP strains. After 4 days of antibiotic induction, the expression levels of virulence genes in CSKP exhibited minimal changes, whereas CRKP showed a marked decrease in *iutA* expression alongside a significant increase in resistance gene expression, particularly *SHV* and *KPC*. By day seven of induction, *rmpA* expression in CSKP continued to rise, while CRKP demonstrated a slight recovery in *iutA* expression compared to day four, although it remained lower than in the original strains. By day ten of induction, *rmpA* expression in CSKP showed a slight increase, while *rmpA2* expression in CRKP increased significantly, with *iutA* expression stabilizing and *KPC* and *SHV* expression rising dramatically compared to the original strains.

According to the research by Hu et al. (2024) on the pathogenic mechanisms of *Klebsiella pneumoniae*, *rmpA* and *rmpA2* are likely involved in various key physiological processes in CSKP and have significant importance in maintaining bacterial structural integrity and virulence. Their higher expression may be closely related to the composition and functional regulation of the outer membrane, enhancing the bacteria’s ability to withstand external environmental pressures, including a certain level of antibiotic resistance (Sun et al., 2022). This might represent an adaptive strategy that CSKP has developed over time. Pountain et al. (2024) highlighted in their studies on bacterial gene regulation that different strains may possess unique regulatory networks. There could be specific transcriptional regulatory mechanisms in CSKP that keep *rmpA* and *rmpA2* in a relatively active transcriptional state. For instance, there may be positive regulatory factors binding specifically to the promoter regions of *rmpA* and *rmpA2*, promoting efficient transcription (He et al., 2023).

The continued increase in *rmpA* expression throughout the induction period may represent an adaptive response by CSKP. In the presence of antibiotics, CSKP may sense cellular stress and upregulate *rmpA* expression to enhance its defensive capabilities. *RmpA* could be involved in the repair mechanisms against antibiotic damage or may improve antibiotic tolerance by modulating the structure and function of the outer membrane (Memar et al., 2020). By day ten, the slight increase in *rmpA* expression may indicate that CSKP has reached a new equilibrium, where the expression level of *rmpA* is sufficient to meet its survival and functional needs in an antibiotic environment.

The significant decline in *iutA* expression in CRKP may be a strategy for adapting to the antibiotic environment. *IutA* may be associated with certain antibiotic-sensitive metabolic pathways or cellular functions, and reducing its expression could lessen CRKP’s dependency on these potentially vulnerable aspects, thereby enhancing its chances of survival. For example, since *iutA* is involved in iron uptake, which can sometimes increase bacterial sensitivity to antibiotics, CRKP may lower its iron acquisition by reducing *iutA* expression, thus decreasing its susceptibility (Sheldon et al., 2016). The antibiotic induction likely triggered a reprogramming of the gene regulatory network within CRKP. Transcriptional regulators may sense the threat posed by antibiotics and initiate a suite of regulatory programs to suppress the expression of genes, like *iutA*, that may be detrimental to survival while activating the transcription of resistance genes. This remodeling of gene expression is a critical mechanism by which CRKP adapts to an antibiotic environment, reflecting the flexibility of bacterial gene regulatory systems and their rapid response capabilities to environmental changes (Hu et al., 2024).

Additionally, throughout the 10 days of antibiotic induction, the expression of resistance genes such as *KPC* and *SHV* significantly increased. A lateral comparison of the induction capabilities of four antibiotics, along with the agar dilution determination of MIC, showed that the expression of resistance genes under polymyxin B induction was not as strong as that under the other three antibiotics, consistent with previous discussions (Yang et al., 2023). The substantial increase in resistance genes like *KPC* and *SHV* further enhances the resistance capabilities of CRKP. As antibiotic pressure persists, CRKP must continuously strengthen its resistance to ensure survival. The high expression of resistance genes enables CRKP to more effectively combat antibiotics, thereby improving its competitive survival in antibiotic environments and ensuring the bacteria’s survival and reproduction in adverse conditions (Budia-Silva et al., 2024).

This study acknowledges several limitations, including the relatively small sample sizes of 50 CSKP and 50 CRKP strains, which may not fully encapsulate the genetic diversity of *Klebsiella pneumoniae*. Additionally, the research being conducted at a single institution may limit the generalizability of the findings. The absence of longitudinal data restricts our ability to observe the evolution of resistance and virulence traits over time. Moreover, while focusing on specific virulence and resistance genes provides valuable insights, the lack of functional studies assessing their direct impacts on pathogenicity may overlook other vital contributing factors. Future research should strive to overcome these limitations to yield more comprehensive insights into the challenges posed by *Klebsiella pneumoniae*.

## 5 Conclusion

This study sheds light on the complex relationship between virulence factors and antibiotic resistance in *Klebsiella pneumoniae*, highlighting that while CSKP strains exhibit higher expression of virulence genes, CRKP strains are becoming increasingly resilient through enhanced expression of resistance genes at the cost of certain virulence traits. The dynamics of gene expression reveal that *Klebsiella pneumoniae* responds to antibiotic inhibition with a marked plasticity, navigating between virulence and resistance in a rapidly changing environment. Continuous monitoring and investigation into these aspects are vital for the development of effective treatment strategies and the management of infections caused by *Klebsiella pneumoniae*.

## Data Availability

The original contributions presented in the study are publicly available. This data can be found here: http://datadryad.org/stash/share/AGJndLDLl3cB9-Nfe38vRs4OmaLXL13njGd3zOR-MKk.
