# Supplementary material for: The impact of antibiotic induction on virulence and antibiotic resistance in Klebsiella pneumoniae: a comparative study of CSKP and CRKP strains
Source: Front Microbiol. 2024 Oct 21;15:1498779. doi: 10.3389/fmicb.2024.1498779 (PMC11532078; doi:10.3389/fmicb.2024.1498779)
Supplement: Supplementary file 1 [file Table_1.DOC]

**Supplemental material**

**Supplementary Table1 Drug susceptibility test**

| **Medicine** |  | **CSKP(n=50)** | |  | **CRKP(n=50)** | |
| --- | --- | --- | --- | --- | --- | --- |
|  |  | **n** | **（%）** |  | **n** | **（%）** |
| Amikacin |  | 2 | 4.0 |  | 23 | 46.0 |
| Amoxicillin/clavulanic acid |  | 16 | 32.0 |  | 50 | 100 |
| Ceftazidime |  | 6 | 12.0 |  | 49 | 98.0 |
| Cefoperazone/Sulbactam |  | 5 | 10.0 |  | 50 | 100 |
| Ceftriaxone |  | 8 | 16.0 |  | 50 | 100 |
| Cefoxitin |  | 3 | 6.0 |  | 46 | 92.0 |
| Cefepime |  | 5 | 10.0 |  | 49 | 98.0 |
| Imipenem |  | 0 | 0 |  | 50 | 100 |
| Levofloxacin |  | 5 | 10.0 |  | 37 | 74.0 |
| Cotrimoxazole |  | 11 | 22.0 |  | 23 | 46.0 |
| Piperacillin/Tazobactam |  | 8 | 16.0 |  | 50 | 100 |
| Ertapenem |  | 0 | 0 |  | 44 | 88.0 |

**Supplementary Table 2** Sequences of the pcr primers

| **Gene** | **Primer sequences(5’-3’)** | **Product（bp）** | **References** |
| --- | --- | --- | --- |
| *rmpA* | F: ACTGGGCTACCTCTGCTTCA | 516 | Lin et al. (2020) |
|  | R: CTTGCATGAGCCATCTTTCA |  |  |
| *rmpA2* | F: CTTTATGTGCAATAAGGATGTT | 451 | Zhang et al. (2022) |
|  | R: CCTCCTGGAGAGTAAGCATT |  |  |
| *iucA* | F：AATCAATGGCTATTCCCGCTG | 239 | Du et al. (2022) |
|  | R：CGCTTCACTTCTTTCACTGACAGG |  |  |
| *iutA* | F: ACCTGGGTTATCGAAAACGC | 1115 | Liu et al. (2019) |
|  | R: GATGTCATAGCCTGATTGC |  |  |
| *iroB* | F：ATCTCATCATCTACCCTCCGCTC | 235 | Zhang et al. (2022) |
|  | R：GGTTCGCCGTCGTTTTCAA |  |  |
| *peg-344* | F：CTTGAAACTATCCCTCCAGTC | 508 | Du et al. (2022) |
|  | R：CCAGCGAAAGAATAACCCC |  |  |
| *ybtS* | F: GACGGAAACAGCACGGTAAA | 242 | Zhou et al. (2020) |
|  | R: GAGCATAATAAGGCGAAAGA |  |  |
| *KPC-2* | F: ATGTCACTGTATCGCCGTCT | 920 | Liu et al. (2021) |
|  | R: TTTTCAGAGCCTTACTGCCC |  |  |
| *NDM-1* | F: CAGCACACTTCCTATCTC | 292 | Liu et al. (2021) |
|  | R: CCGCAACCATCCCCTCTT |  |  |
| *CTX-M-1* | F: GGCCCATGGTTAAAAAATCACTGC | 944 | Lahlaoui al. (2014) |
|  | R: CAGCGCTTTTGCCGTCTAAG |  |  |
| *CTX-M3* | F: GTTGTTGTTATTTCGTATCTTCC | 934 | Lahlaoui al. (2014) |
|  | R: CGATAAACAAAAACGGAATG |  |  |
| *SHV* | F: GCCTTTATCGGCCTTCACTCAAG | 972 | Liu et al. (2021) |
|  | R: TTAGCGTTGCCAGTGCTCGATCA |  |  |
| *DHA* | F: CTGATGAAAAAATCGTTATC | 898 | Liu et al. (2021) |
|  | R: ATTCCAGTGCACTCAAAATA |  |  |
| *16SRNA* | F: AGAGTTTGATCTTGGCTCAG | - | Jiang et al. (2024) |
|  | R: GGTTACCTTGTTACGACTT |  |  |
| *q-rmpA* | F: AGGGAAATGGGGAGGGTACAAAATG | 78 | Jiang et al. (2024) |
|  | R: CCCGAAACGTCAAGCCACATC |  |  |
| *q-rmpA2* | F: AGTCAATGGATGTGGCTTGAC | 71 | Jiang et al. (2024) |
|  | R: ATGTCATAATCACACCCTTGAGG |  |  |
| *q-iucA* | F: TGAAAGAAGTGAAGCGTGGAATGC | 77 | Jiang et al. (2024) |
|  | R: GAAGCGAGCCTGTAGCGTCTG |  |  |
| *q-iutA* | F: ACATCCGCCGACGCCATTC | 120 | Jiang et al. (2024) |
|  | R: CACGCCCTGGGAGAAGTTGAG |  |  |
| *q-KPC* | F: AAACTGACACTGGGCTCTGC | 135 | Li et al.(2021) |
|  | R: TCCGGTTTTGTCTCCGACTG |  |  |
| *q-SHV* | F: AGCCGCTTGAGCAAATTAAA | 77 | Yengui et al.(2022) |
|  | R: GCTGGCCAGATCCATTTCTA |  |  |

**References**

1.Lin, Z. W., Zheng, J. X., Bai, B., Xu, G. J., Lin, F. J., Chen, Z., et al. (2020). Characteristics of Hypervirulent *Klebsiella pneumoniae*: Does Low Expression of rmpA Contribute to the Absence of Hypervirulence?. *Frontiers in microbiology*, 11, 436. https://doi.org/10.3389/fmicb.2020.00436.

2.Zhang, Y., Xu, Y., & Huang, Y. (2022). Virulence Genotype and Correlation of Clinical Severeness with Presence of the Type VI Secretion System in *Klebsiella pneumoniae* Isolates Causing Bloodstream Infections. I*nfection and drug resistance*, 15, 1487–1497. https://doi.org/10.2147/IDR.S353858.

3.Du, Q., Pan, F., Wang, C., Yu, F., Shi, Y., Liu, W., Li, Z., et al. (2022). Nosocomial dissemination of hypervirulent *Klebsiella pneumoniae* with high-risk clones among children in Shanghai. *Frontiers in cellular and infection microbiology*, 12, 984180. https://doi.org/10.3389/fcimb.2022.984180.

4.Liu, Y., Du, F. L., Xiang, T. X., Wan, L. G., Wei, D. D., Cao, X. W., et al. (2019). High Prevalence of Plasmid-Mediated Quinolone Resistance Determinants Among Serotype K1 Hypervirulent *Klebsiella pneumoniae* Isolates in China. *Microbial drug resistance (Larchmont, N.Y.)*, 25(5), 681–689. https://doi.org/10.1089/mdr.2018.0173.

5. Zhou, M., Lan, Y., Wang, S., Liu, Q., Jian, Z., Li, Y., et al. (2020). Epidemiology and molecular characteristics of the type VI secretion system in *Klebsiella pneumoniae* isolated from bloodstream infections. *Journal of clinical laboratory analysis*, 34(11), e23459. https://doi.org/10.1002/jcla.23459.

6. Liu, S., Wang, X., Ge, J., Wu, X., Zhao, Q., Li, Y.M., et al. (2021). Analysis of Carbapenemase-Resistant Genotypes of Highly Virulent *Klebsiella pneumoniae* and Clinical Infection Characteristics of Different MLST Types. *Evid Based Complement Alternat Med*, 3455121. doi:10.1155/2021/3455121.

7. Lahlaoui, H., Ben Haj Khalifa, A., & Ben Moussa, M. (2014). Epidemiology of Enterobacteriaceae producing CTX-M type extended spectrum β-lactamase (ESBL). *Medecine et maladies infectieuses*, 44(9), 400–404. https://doi.org/10.1016/j.medmal.2014.03.010.

8. Jiang, M., Qiu, X., Shui, S., Zhao, R., Lu, W., Lin, C., et al. (2024). Differences in molecular characteristics and expression of virulence genes in carbapenem-resistant and sensitive *Klebsiella pneumoniae* isolates in Ningbo, China. *Frontiers in microbiology,* 15, 1356229. https://doi.org/10.3389/fmicb.2024.1356229.

9. Li, B., & Yan, T. (2021). Next generation sequencing reveals limitation of qPCR methods in quantifying emerging antibiotic resistance genes (ARGs) in the environment. *Applied microbiology and biotechnology*, 105(7), 2925–2936. https://doi.org/10.1007/s00253-021-11202-4.

10.Yengui, M., Trabelsi, R., Khannous, L., Mathlouthi, N. E., Adnan, M., Siddiqui, A. J., et al. (2022). Rapid Detection of Beta-Lactamases Genes among Enterobacterales in Urine Samples by Using Real-Time PCR. *BioMed research international*, 2022, 8612933. https://doi.org/10.1155/2022/8612933.

**Supplementary Table 3 List of original strains selected for antibiotic induction experiment and RT-qpcr**

| **Group** | **Strain** | ***rmpA*** | ***rmpA2*** | ***iucA*** | ***iutA*** | *iroB* | *peg-344* | *ybtS* | *KPC-2* | *NDM-1* | *CTX-M-1* | *CTX-M3* | *SHV* | *DHA* |
| --- | --- | --- | --- | --- | --- | --- | --- | --- | --- | --- | --- | --- | --- | --- |
| CSKP | 3 | **+** | **+** | **+** | **+** | **+** | **+** | **+** | **/** | **/** | **/** | **/** | **/** | **/** |
| CSKP | 22 | **+** | **-** | **+** | **+** | **+** | **+** | **+** | **/** | **/** | **/** | **/** | **/** | **/** |
| CSKP | 27 | **+** | **+** | **+** | **+** | **+** | **+** | **-** | **/** | **/** | **/** | **/** | **/** | **/** |
| CSKP | 28 | **+** | **+** | **-** | **-** | **+** | **+** | **+** | **/** | **/** | **/** | **/** | **/** | **/** |
| CSKP | 31 | **+** | **+** | **+** | **+** | **-** | **+** | **+** | **/** | **/** | **/** | **/** | **/** | **/** |
| CSKP | 36 | **-** | **+** | **+** | **+** | **+** | **+** | **+** | **/** | **/** | **/** | **/** | **/** | **/** |
| CSKP | 42 | **+** | **+** | **+** | **+** | **+** | **+** | **-** | **/** | **/** | **/** | **/** | **/** | **/** |
| CSKP | 45 | **+** | **+** | **+** | **+** | **+** | **+** | **+** | **/** | **/** | **/** | **/** | **/** | **/** |
| CRKP | 51 | - | - | **-** | **-** | **-** | **-** | **+** | **+** | **-** | **-** | **-** | **+** | **+** |
| CRKP | 52 | - | + | **+** | **+** | **-** | **-** | **+** | **+** | **-** | **-** | **-** | **+** | **-** |
| CRKP | 55 | - | - | **-** | **+** | **-** | **-** | **+** | **-** | **+** | **-** | **-** | **+** | **-** |
| CRKP | 58 | - | - | **-** | **-** | **-** | **-** | **+** | **+** | **-** | **-** | **+** | **+** | **-** |
| CRKP | 61 | + | + | **+** | **+** | **+** | **+** | **+** | **+** | **-** | **-** | **-** | **+** | **-** |
| CRKP | 80 | + | + | **+** | **+** | **-** | **-** | **+** | **+** | **-** | **-** | **+** | **+** | **-** |
| CRKP | 85 | + | + | **-** | **+** | **-** | **+** | **+** | **+** | **-** | **-** | **+** | **+** | **-** |
| CRKP | 86 | + | + | **+** | **+** | **-** | **+** | **+** | **+** | **-** | **-** | **+** | **+** | **-** |

**Supplementary Table 4** List of the MIC of the four antibiotics in the original strains

| **Group** | **Strain** | Ciprofloxacin | Imipenem | Polymyxin B | Ceftazidime-avibactam |
| --- | --- | --- | --- | --- | --- |
| CSKP | 3 | 0.5 | 0.5 | 0.125 | 0.25 |
| CSKP | 22 | 2 | 0.125 | 0.125 | 0.125 |
| CSKP | 27 | 1 | 0.25 | 0.0625 | 0.125 |
| CSKP | 28 | 1 | 0.125 | 0.125 | 0.125 |
| CSKP | 31 | 1 | 0.5 | 0.125 | 0.125 |
| CSKP | 36 | 0.5 | 0.5 | 0.0625 | 0.125 |
| CSKP | 42 | 1 | 0.5 | 0.0625 | 0.125 |
| CSKP | 45 | 1 | 0.25 | 0.0625 | 0.125 |
| CRKP | 51 | 4 | 16 | 0.0625 | 0.5 |
| CRKP | 52 | >64 | >64 | 0.0625 | 0.125 |
| CRKP | 55 | >64 | >64 | 0.0625 | 0.25 |
| CRKP | 58 | 2 | 16 | 0.0625 | 0.125 |
| CRKP | 61 | 1 | 64 | 0.0625 | 0.5 |
| CRKP | 80 | 1 | >64 | 0.0625 | 0.5 |
| CRKP | 85 | >64 | >64 | 0.0625 | 0.5 |
| CRKP | 86 | >64 | >64 | 0.0625 | 0.125 |
